# SARS-CoV-2 Spike Pseudoviruses: A Useful Tool to Study Virus Entry and Address Emerging Neutralization Escape Phenotypes

**DOI:** 10.3390/microorganisms9081744

**Published:** 2021-08-16

**Authors:** Raj Kalkeri, Zhaohui Cai, Shuling Lin, John Farmer, Yury V. Kuzmichev, Fusataka Koide

**Affiliations:** 1 Department of Infectious Disease Research, Drug Development Division, Southern Research, 431 Aviation Way, Frederick, MD 21701, USA; zcai@southernresearch.org (Z.C.); slin@southernresearch.org (S.L.); ykuzmichev@southernresearch.org (Y.V.K.); 2 Department of Infectious Disease Research, Drug Development Division, Southern Research, 2000 Ninth Avenue South, Birmingham, AL 35205, USA; jfarmer@southernresearch.org

**Keywords:** COVID-19, SARS-CoV-2, pseudovirus, variants of concern, neutralizing antibodies, PBNA, lentiviral vector, MLV vector, spike, neutralization assay, antibody

## Abstract

SARS-CoV-2 genetic variants are emerging around the globe. Unfortunately, several SARS-CoV-2 variants, especially variants of concern (VOCs), are less susceptible to neutralization by the convalescent and post-vaccination sera, raising concerns of increased disease transmissibility and severity. Recent data suggests that SARS-CoV-2 neutralizing antibody levels are a reliable correlate of vaccine-mediated protection. However, currently used BSL3-based virus micro-neutralization (MN) assays are more laborious, time-consuming, and expensive, underscoring the need for BSL2-based, cost-effective neutralization assays against SARS-CoV-2 variants. In light of this unmet need, we have developed a BSL-2 pseudovirus-based neutralization assay (PBNA) in cells expressing the human angiotensin-converting enzyme-2 (hACE2) receptor for SARS-CoV-2. The assay is reproducible (R^2^ = 0.96), demonstrates a good dynamic range and high sensitivity. Our data suggest that the biological Anti-SARS-CoV-2 research reagents such as NIBSC 20/130 show lower neutralization against B.1.351 SA (South Africa) and B.1.1.7 UK (United Kingdom) VOC, whereas a commercially available monoclonal antibody MM43 retains activity against both these variants. SARS-CoV-2 spike PBNAs for VOCs would be useful tools to measure the neutralization ability of candidate vaccines in both preclinical models and clinical trials and would further help develop effective prophylactic countermeasures against emerging neutralization escape phenotypes.

## 1. Introduction

Severe acute respiratory syndrome coronavirus 2 (SARS-CoV-2), the causative agent of coronavirus disease 2019 (COVID-19), remains a major global health challenge that has been responsible for more than 4 million deaths since the start of the pandemic in December 2019, with an estimated 187 million confirmed cases as of 12 July 2021 [1]. While the development of novel prophylactic and therapeutic measures, such as vaccines, monoclonal antibodies, and antiviral medications, has been instrumental in slowing down the pace of the pandemic, the emergence of several variants of concern (VOCs) has raised issues about potential immune escape [2]. SARS-CoV-2 is an enveloped virus with a positive-sense single-stranded RNA genome. It belongs to the β-coronavirus genus of the *Coronaviridae* family [3]. Spike (S) glycoprotein, present on the virion surface, is responsible for the virus entry into the susceptible cells by attachment to the human Angiotensin-Converting Enzyme 2 (hACE2) receptor. Due to the immunogenic nature of S glycoprotein [4,5,6,7], it is used as a component of multiple vaccines. Measuring the effect of neutralizing antibodies, an important correlate of protection, against the S glycoprotein is of primary importance in fighting the pandemic. Currently, a number of clinical trials investigating such therapeutic interventions are ongoing [8].

In order to determine the efficacy of approaches for measuring the effect of neutralization against emerging VOC, assays that are capable of measuring serological responses to the spike glycoprotein are of extreme importance. Current assays used for such purposes rely on principles of microneutralization (MN) or enzyme-linked immunosorbent assays (ELISA) and several ELISA derivatives [9,10,11]. SARS-CoV-2 MN assays relying on the neutralization of a wild-type, replicating virus are considered the gold-standard methods for the evaluation of the neutralization ability of coronavirus-induced antibodies. However, in its present form, the MN assay is expensive and labor-intensive, requiring the use of a biosafety level three containment (BSL-3), and making it challenging to adapt in large-scale clinical trials [12]. At the other extreme are ELISA methods. While they are considered safe and adaptable to a high-throughput format, they measure total antibodies against the protein and do not measure the neutralization titers, in contrast with MN formats [10,13,14,15]. 

To avoid the use of a highly restrictive BSL-3 environment and improve upon the ELISA, the implementation of replication-deficient pseudoviruses containing the viral coat proteins of interest has been suggested as a safe and useful alternative [16]. Pseudovirus-based platforms have been successfully employed in the study of highly infectious and pathogenic viruses, including Ebola, Middle Eastern Respiratory Syndrome (MERS), Rabies, Marburg, Lassa, and others [17,18,19,20,21]. Recently, a number of groups have successfully generated SARS-CoV-2 pseudoviruses using murine leukemia virus (MLV), vesicular stomatitis virus (VSV), as well as human immunodeficiency virus (HIV) platforms and employed them for the evaluation of neutralizing antibodies through different readout systems [22,23,24,25,26,27,28]. However, there is currently limited data on the use of such systems in the evaluation of the SARS-CoV-2 VOC. To address this, we developed and optimized a robust pseudovirus-based neutralization assay (PBNA) and evaluated it against SARS-CoV-2 614D and two VOC-B.1.1.7, UK (United Kingdom) variant, and B.1.351, SA (South Africa) variant. The objective of this pseudovirus neutralization assay (PBNA) is to establish a standardized assay for use in the United States Food and Drug Administration (FDA) mandated preclinical safety studies using the non-human primate (NHP) SARS-CoV-2 infection models, such as rhesus macaques (RM), cynomolgus monkeys (CM), and African green monkeys (AGMs). This application advances the field by providing an assay to allow for the comparison of NHP cross-species specific SARS-CoV-2 neutralizing responses, and subsequent data may be used to validate the concordance of non-clinical and clinical neutralizing antibody responses.

Furthermore, we evaluated the performance characteristics of the positive plasma control (NIBSC 20/130) and two commercially available monoclonal antibodies in the PBNA. Here, we present the detailed methods and the performance of the PBNA, which could be adapted for use in various quantitative, medium/high-throughput virus neutralization screens in a standard BSL-2 laboratory environment. 

## 2. Materials and Methods

### 2.1. Pseudoviruses

The virus backbone (HIV-1 NL4-3 ΔEnv Vpr Luciferase Reporter Vector, pNL4-3.Luc.R-E-, NIH AIDS Reagent Program, Catalog Number: 3418) was licensed and obtained from the New York University School of Medicine (New York University School of Medicine, New York, NY, USA). A codon-optimized SARS-CoV-2 spike gene from isolate 2019-nCoV_HKU-SZ-002a_2020 (GenBank: MN938384) was synthesized at GeneWiz (GeneWiz, South Plainfield, NJ, USA). The spike gene was cloned into eukaryotic expression plasmid pcDNA3.1 (Catalog number NR-52420, BEI Resources, Manassas, VA, USA) to generate a plasmid, designated as pSRC332. For pseudovirus production, Lenti-X-293T cells were co-transfected with pNL4-3.Luc.R-E- and pSRC322 using JetPrime Transfection Reagent (Polyplus Transfection, Catalog number 114-15, New York, NY, USA). Briefly, 6 × 10^6^ Lenti-X-293T cells were seeded in a T75 flask one day before transfection. The next day, the cells were co-transfected with 3 μg of pSRC332 and 12 μg of pNL4-3.Luc.R-E- using JetPrime Transfection Reagent following the manufacturer’s instruction. Five hours later, the plasmid DNA-transfection complexes were removed, and the cells were washed with PBS once, and then a new culture medium was added. Seventy-two hours post-transfection, the SARS-CoV-2 pseudoviruses containing culture supernatant were harvested and clarified by centrifugation at 1200 RPM for 5 min and stored at −70 °C in 0.5 mL aliquots until use. A set of replication-deficient murine leukemia virus (MLV)-based pseudoviruses for the 614D variant (Catalog number MBS434275), B.1.351 SA variant (Catalog number MBS434287), and B.1.1.7 UK variant (Catalog number MBS434286) were purchased from MyBioSource (MyBioSource Inc., San Diego, CA, USA). 

### 2.2. Serum and Monoclonal Antibodies

NIBSC 20/130 (National Institute of Biological Standards and Control, Blanche Lane, Ridge, Herts, UK), anti-SARS-CoV-2 RBD neutralizing antibody, human IgG1 (Catalog number SAD-S35, Acro Biosystems, Newark, DE, USA), and SARS-CoV-2 (2019-nCoV) spike neutralizing mouse monoclonal antibody (Catalog number 40591-MM43, Sino Biological, Wayne, PA, USA) were used as positive controls in the assay. Sera from unvaccinated healthy monkeys were used as negative controls. Sera 28-days post-infection from three (3) different species of NHPs, rhesus macaques (RM), cynomolgus monkeys (CM), and African green monkeys (AGMs) (*n* = 3 each), challenged with SARS-CoV-2 (SARS-CoV-2 USA_WA1/2020 strain at 4.0 × 10^6^ TCID_50_/mL through intra-tracheal route) were used in the pseudovirus assay. In vivo study design was reviewed by the IACUC at Southern Research Institute and was approved on 06/10/2020; it was assigned IACUC tracking number 20-06-021B.

### 2.3. Pseudovirus-Based Neutralization Assay

HEK293T-hACE2 cells (NR-52511, BEI Resources, Manassas, VA, USA), used for the assay, were cultured using Dulbecco’s Minimal Essential Medium (DMEM) (Catalog number BE12- 604Q, Lonza, Walkersville, MD, USA) with 10% fetal bovine serum (FBS) (Catalog number PS-FB1, Peak Serum, Wellington, CO, USA). The cells were subcultured twice a week at a split ratio of 1:5 to 1:10 using standard cell culture techniques and the cell culture media. Total cell numbers and percent viability were determined by trypan blue dye exclusion using a hemocytometer. Cell batches with greater than 95% cell viability were utilized for the assays. On the day before the assay, 2 × 10^4^ HEK293T-hACE2 cells were seeded into each well of 96-well plates in 100 µL volume of DMEM with 10% FBS without antibiotics. The next day, the test serum, heat-inactivated at 56 °C for 30 min, was serially diluted 5-fold in DMEM with 2% FBS, followed by mixing with an equal volume of SARS-CoV-2 pseudoviral particles (final volume of 100 μL) and incubation at 37 °C for 1 h. Media from the cell culture was removed, and 100 µL of the serum-virus mixture was added into each well. Each assay plate contained cells without pseudovirus infection (cell control/background) and pseudovirus-only control (virus control). Each assay run also included a positive control (serum or antibody with known neutralizing activity). The cell culture plate was centrifuged at 700 RPM for 15 min at 4°C, followed by incubation for 72 h at 37 °C with 5% CO_2_. Luciferase activity in the infected cells was measured by removing the culture supernatant and adding 50 µL if luciferase assay reagent (Firefly luciferase reagent, Promega. Madison, WI, USA). Luminescence was recorded using the luminescence plate reader (Clariostar plate reader, BMG Labtech Inc, Cary, NC, USA). After background subtraction, the luminescence signal was normalized to the virus control and analyzed using GraphPad Prism version 9.1.1 for Windows (GraphPad Software, San Diego, CA, USA) with a four-parameter logistic (4-PL) curve fitting to calculate the 50% pseudovirus-based neutralization index (PBNI_50_). The pseudovirus neutralization assay assesses neutralization activity by calculating a pseudovirus neutralization index 50% (PBNI_50_). Negative control sera, positive sera, and positive control monoclonal antibodies (MABs) were diluted by 5-fold, plated in triplicate wells, and serially diluted by performing six 1:5 in-plate dilutions. Dilution ranges and concentrations for sera and MABs were 10 to 31,500-fold and 160-to 5 × 10^6^-fold. The relative luminescence unit (RLU) values of sera and MABs were normalized by subtracting the mean RLU of the uninfected cell control. The pseudovirus neutralization index was calculated by GraphPad Prism 9 software using the mean RLU values at each dilution and applying a 4-PL regression model to calculate the IC_50_, defined as a 50% reduction in the RLU calculated from the curve equation below, with the parameter of *y* = 50:

(1) $$x=c{\left(\frac{a-d}{y-d}-1\right)}^{\frac{1}{b}}$$

The rationale for the calculation of the PBNI_50_ was to reduce bias and allow normalization in the statistical comparison of the neutralization activity of different controls and samples. Use of the arithmetically calculated 50% inhibition titer introduces bias and limits the inter-sample neutralization activity comparison as the maximum signal, minimum signal, and slope of the sigmoid antibody response is not defined, nor can confidence limits be set.

## 3. Results

PBNA for SARS-CoV-2 spike protein variants (614D, B.1.1.7 UK, and B.1.351 SA) was developed in HEK293T-cells expressing the hACE2 receptor for SARS-CoV-2. A set of positive controls (NIBSC 20/130, Acro-SAD-S35, and Sino-40591-MM43) were tested in this assay system. The results demonstrate that NIBSC 20/130 shows significantly reduced neutralization against the B.1.351 SA and B.1.1.7 UK variants, whereas Sino-40591-MM43 shows better neutralization against these variants.

### 3.1. Dose Dependence of SARS-CoV-2 Spike Protein Pseudovirus Variants

**Figure 1:**
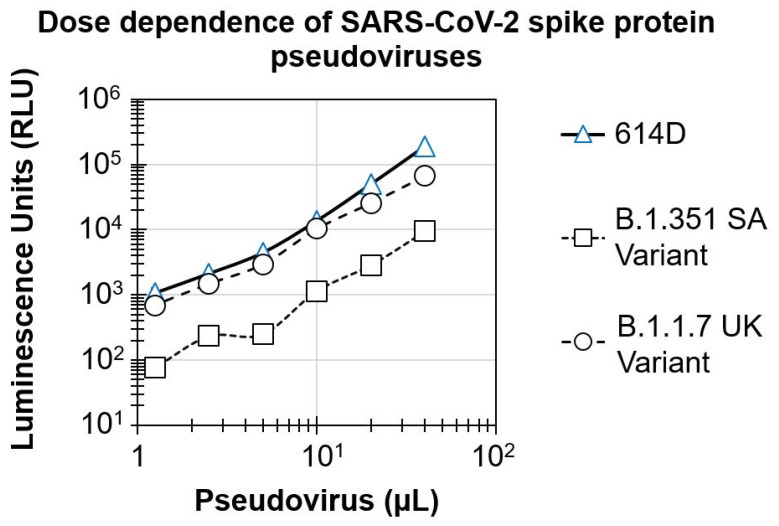
Dose dependence of SARS-CoV-2 spike protein pseudoviruses: HEK293T-hACE2 cells seeded in 96-well plates were infected with increasing concentrations of SARS-CoV-2 spike protein pseudoviruses. Intracellular luciferase signal (as a marker of spike protein-mediated pseudovirus infection) measured at the end of 3 days is shown on the *Y*-axis. Triangles–614D variant, Circles–B.1.1.7 UK variant, squares boxes–B.1.351 SA variant.

To develop the pseudovirus-based assay, HEK293T-hACE2 cells were infected with increasing concentrations of SARS-CoV-2 spike protein pseudovirus variants. The luciferase signal in the infected cells was analyzed after 3 days post-infection. As shown in Figure 1, there was a dose-dependent increase in the signal for all three pseudoviruses, with a dynamic range of 2-logs. Pseudoviruses for the 614D and B.1.1.7 UK variant showed similar luciferase signal (signal of 188,869.5 ± 13,500.8 and 66,315 ± 1640.5 RLU, respectively) at the highest concentration tested (40 µL/well). This resulted in a robust signal to the background (S/B) ratio in the range of 2337 to 6656. In contrast, cells infected with the B.1.351 SA variant showed a significantly lower signal (9400.5 RLU, 1-log lower) compared to the 614D and B.1.1.7 UK variant pseudoviruses, at the highest concentration tested. Based upon these results and the optimal S/B level, we chose the virus inoculum for the 614D, B.1.1.7 UK and B.1.351 SA variants for testing the serum samples to detect neutralizing antibodies.

### 3.2. Suitability of PBNA for Evaluation of Neutralization Antibodies and Reproducibility of the SARS-CoV-2 Spike Protein Pseudovirus Assay

It is important for the assays to be reproducible between different experiments to consistently determine the neutralizing antibodies in the test serum samples. To address this, we tested six serum samples collected 28-days post-infection from different species of monkeys (rhesus macaques, cynomolgus monkeys, African green monkeys, *n* = 2 each) challenged with SARS-CoV-2 in the PBNA as mentioned in Section 2. Serum samples from all three monkey species showed PBNI_50_ ranging from 147 to 732.7. Human IgG1Anti-SARS-CoV-2 spike RBD neutralizing antibody (Acro-SAD-S35) and a mouse monoclonal antibody SARS-CoV-2 (2019-nCoV) spike neutralizing antibody (Sino-40591-MM43) were used as positive controls in the assay. Non-challenged monkey serum was used as a negative control in the assay. The positive controls showed robust inhibition of the pseudovirus infection in the PBNA, with PBNI_50_ of 11,164.0 and 15,706.0 respectively. The negative control showed a PBNI_50_ of 123.3 ± 54.2 in the assay, which might be due to preexisting antibodies (possibly due to prior exposure to other coronaviruses). More than the 2-log difference between the negative and positive controls confirm the significant assay dynamic range, which could enable screening antibodies with the ability to differentiate between samples with varying neutralization potencies against SARS-CoV-2. 

**Figure 2:**
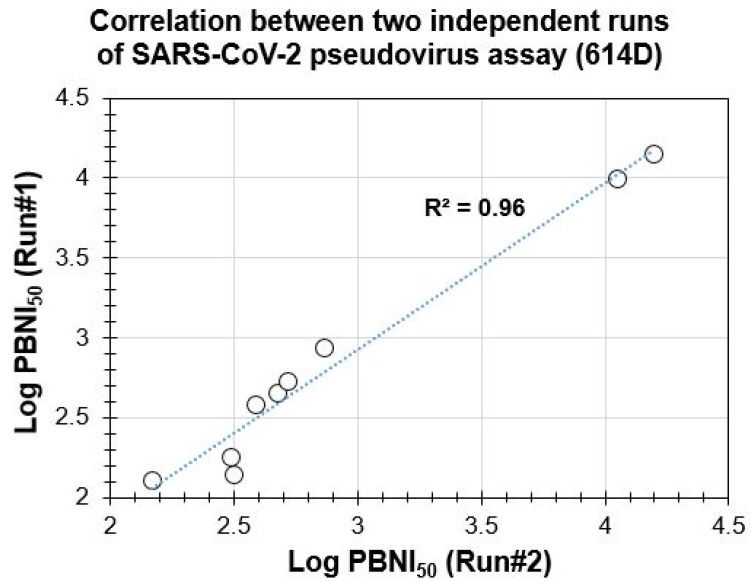
Reproducibility of the SARS-CoV-2 spike protein pseudovirus-based neutralization assay: PBNA for 6 serum samples from SARS-CoV-2-challenged NHP, 2 positive and 1 unchallenged NHP controls was conducted in two independent experiments (11 and 18 June 2021). Log transformed PBNI_50_ from each experiment is plotted on the *X*- and *Y*-axis, respectively. The correlation of R^2^ = 0.96, with the trend line between the PBNI_50_ from both the experiments, is shown by the dotted line in the figure.

The assay was repeated on two different days by the same operator. Data from both experiments were compared and are shown in Figure 2. The positive controls showed robust activity in both the assays with a mean ± SD of 10,522.5 ± 907.2 and 14,944 ± 1077.6 in the assay. Both sets of data showed strong correlations with an R^2^ value of 0.96, suggesting that the assay is reproducible. The data also suggests that all three monkey species (rhesus macaques, cynomolgus monkeys, African green monkeys) developed neutralizing antibodies (mean ± SD of 298.7 ± 228.9, 313.9 ± 98.8, 665.1 ± 191.7, respectively).

### 3.3. SARS-CoV-2 Challenged NHP Serum Samples Show Neutralization against SARS-CoV-2 614D and B.1.1.7 UK Variants

**Figure 3:**
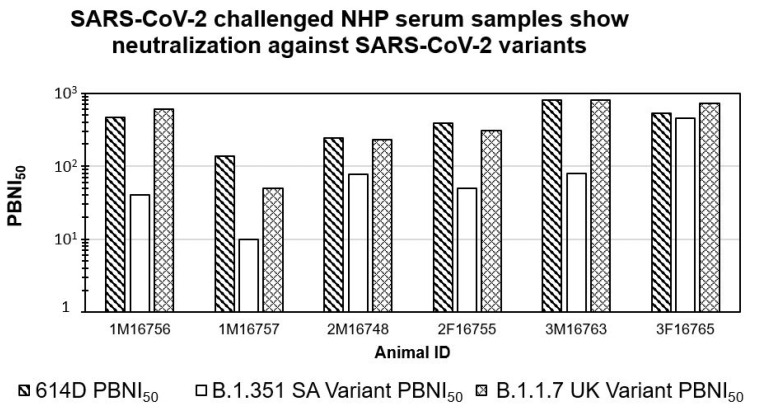
SARS-CoV-2-challenged NHP serum samples show neutralization against SARS-CoV-2 variants: Six (6) serum samples from SARS-CoV-2-challenged NHPs (N = 2 from each species: rhesus macaques (1M16756, 1M16757), cynomolgus monkeys (2M16748, 2F16755), African green monkeys (3M16763, 3F16765)) were subjected to PBNA using the pseudoviruses for 614D and variants as described in Section 2. The PBNI_50_ for each animal is shown on the *Y*-axis. Crosshatch-filled bars show the average PBNI_50_ from two independent experiments for 614D, plain bars show the PBNI_50_ for the B.1.351 SA variant, diamond-filled bars show the PBNI_50_ for the B.1.1.7 UK variant.

Recently SARS-CoV-2 VOC have been reported in the literature. There are some concerns about the differential neutralizing abilities induced by SARS-CoV-2 infection against the latter. To evaluate the neutralizing antibodies against VOC, we tested six serum samples from monkeys challenged with the SARS-CoV-2 USA_WA1/2020 strain against pseudoviruses expressing spike proteins of the B.1.351 SA and B.1.1.7 UK variants in the PBNA. As shown in Figure 3, these serum samples showed similar neutralizing antibodies against both the 614D and B.1.1.7 UK variant. For the B.1.1.7 UK variant, PBNI_50_ ranged from 49.6 to 811.5. Interestingly, the majority of the serum samples (five out of six) showed lower amounts of neutralizing antibodies (PBNI_50_ < 100) against B.1.351 SA. Only one animal (3F16765) showed PBNI_50_ of 449.2 against B.1.351 SA, which was similar to both the 614D and B.1.1.7 UK variant PBNI_50_ levels. These data suggest that neutralizing antibodies elicited by the SARS-CoV-2 USA_WA1/2020 strain are effective against the B.1.1.7 UK variant but less effective against the B.1.351 SA variant.

### 3.4. Linearity of Neutralization Index (PBNI_50_) Generated by the Pseudovirus Assay

**Figure 4:**
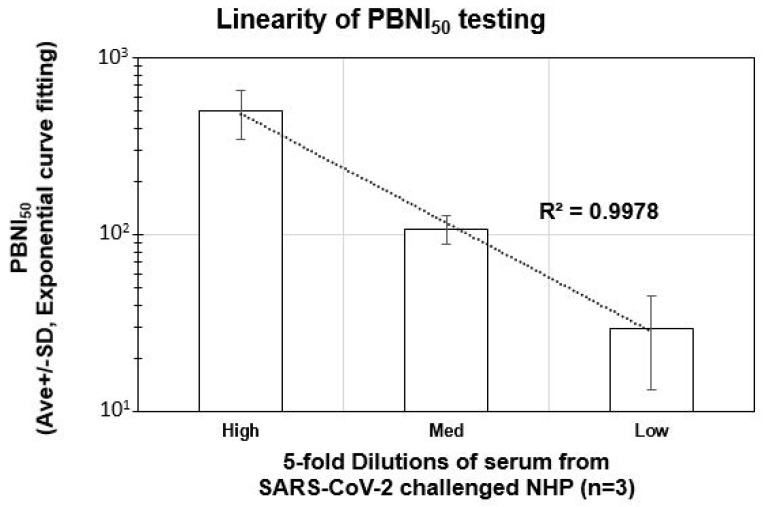
Linearity of PBNI_50_ testing: three (3) serum samples from SARS-CoV-2-challenged NHPs (1M16756, 3M16763, 3F16765) with a high PBNI_50_ were serially diluted 5-fold to generate test samples with a high, medium, and low PBNI_50_, followed by a PBNA with 6-point dilution in triplicate at each dilution. The average ± SD of the three PBNI_50_ for high, medium and low test samples are shown on the *Y*-axis. The *X*-axis denotes the test samples with high, medium, and low PBNI_50_. Trend analysis between the three groups is shown by the dotted line (R^2^ = 0.9978).

In order to identify the linearity of neutralization index (PBNI_50_), three NHP serum samples with high PBNI_50_ (from animals 1M16756, 3M16763, 3F16765) were serially diluted (5-fold) to generate test samples with high, medium, and low PBNI_50_. A positive control (Acro-SAD-S35) similarly diluted was also used as a positive control in the assay. Nine test samples (three each of high, medium, and low PBNI_50_ samples) were tested in the PBNA. The results shown in Figure 4 reveal a linear reduction in the PBNI_50_ titers. High, medium, and low PBNI_50_ test groups showed a PBNI_50_ (mean ± SD) of 504.7 ± 156.3, 108.6 ± 20.2 and 29.5 ± 16.2, respectively. The slope analysis revealed a robust R^2^ value of 0.9978, suggesting a linear relationship between the expected and measured PBNI_50_ values. Data analysis of the positive control also showed a linear range of PBNI_50_ for high, medium, and low PBNI_50_ samples ranging from 6819 to 115.5 with an R^2^ value of 0.989.In addition to the linearity of dilution, this data also revealed the sensitivity of the PBNA with a lower PBNI_50_ detection of 29.5 ± 16.2.

### 3.5. Comparison of Different Positive Controls in PBNA to Evaluate Neutralization against Variants of Concern

**Figure 5:**
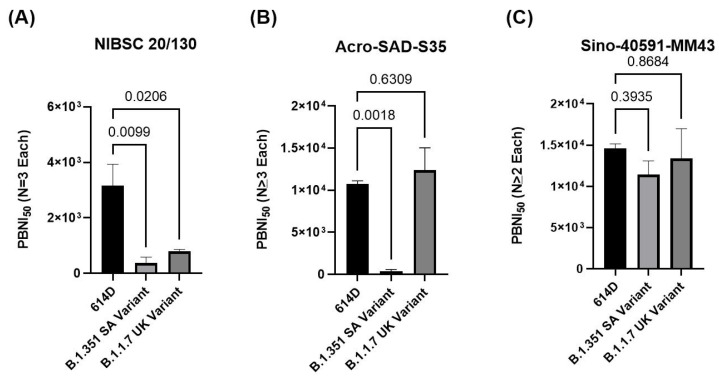
The NIBSC 20/130 control shows lower neutralization against VOCs compared to 614D: Three positive controls (NIBSC 20/130, Acro-SAD-S35, Sino 40591-MM43) were subjected to PBNA using the pseudoviruses for the 614D (**A**), B.1.351 SA (**B**), and B.1.1.7 UK (**C**) variants, as described in Section 2. Ave ± SD of PBNI_50_ from multiple experiments as indicated in the figure are shown on the *Y*-axis. Ordinary one-way analysis (multiple comparisons) was performed using GraphPad Prism version 9.1.1 for Windows; GraphPad Software, San Diego, CA, USA, www.graphad.com (accessed on 15 July 2021) Adjusted *p*-values are shown in the figure.

A universal standard is necessary to harmonize the assays in different laboratories around the globe. Such a standard would facilitate the comparison of assay results from diverse laboratories. Currently, the NIBSC 20/130 research reagent (anti-SARS-CoV-2 antibody) offered by NIBSC is intended to be used as a positive control in assays for the development and evaluation of anti-SARS-CoV-2 antibodies. NIBSC 20/130 was tested in the PBNA against all three pseudoviruses (614D, B.1.351 SA, and B.1.1.7 UK variant) in PBNA. Two other positive controls (Acro-SAD-S35 and Sino-40591-MM43) were also included in the assay. As shown in Figure 5A and Table 1, NIBSC 20/130 demonstrated robust activity against the 614D variant (PBNI_50_ of 3158.3 ± 1344.5), moderate activity against the B.1.1.7 UK variant (PBNI_50_ of 796.4 ± 127.5), and significantly poor activity against the B.1.351 SA variant (PBNI_50_ of < 373.3 ± 369.5). Another positive control, Acro-SAD-S35 (Figure 5B), showed robust activity against both the 614D and B.1.1.7 UK variants (PBNI_50_ of 10,764.5 ± 722.6 and 12,392 ± 4577.1, respectively), but poor activity against the B.1.351 SA variant (PBNI_50_ of < 373.33 ± 369.5). In contrast, the monoclonal antibody Sino-40591-MM43 (Figure 5C) showed robust activity against all three variants; 614D, B.1.351 SA, and B.1.1.7 UK variants (PBNI_50_ of 14,620.3 ± 946, 11.417.7 ± 2912.1, 13,374.5 ± 5118.8, respectively). These data suggest the differential neutralization of the positive controls (NIBSC 20/130, Acro-SAD-S35, and Sino-40591-MM43) against the 614D, B.1.351 SA, and B.1.1.7 UK variants.

## 4. Discussion

Assay dynamic range helps in the appropriate differentiation of the vaccines with differing neutralization potencies. In addition to measuring the 50% inhibition, a wide dynamic range also enables the differentiation of robust inhibition (90–99%) and can help rank the vaccine candidates based on their neutralization profiles. A dose-dependent, 2-log dynamic range in the assay signal observed in our initial dose range testing experiments for all three viruses, along with the robust S/B ratio, confirmed the suitability of this assay, though we consistently observed 1-log lower assay signals in cells infected with B.1.351 SA variant pseudovirus compared to either 614D or B.1.1.7 UK variant. It remains to be seen, however, if the lower signal in the B.1.351 SA variant pseudovirus is either due to lower infection levels or if the assay conditions need to be further fine-tuned to increase the assay signal. However, as we observed a sufficient 2-log dynamic range and S/B ratio needed for the confirmation of neutralization, we proceeded further with the evaluation of the test serum and positive controls.

As the goal of our research was to evaluate the suitability of the PBNA for measuring the neutralization of preclinical samples, serum from six NHPs challenged with the SARS-CoV-2 WA1/2020 strain were tested against the 614D pseudovirus. The test serum samples and the positive controls used in the assay demonstrated dose-dependent inhibition of the assay signal (pseudovirus-based luciferase signal) (Example of dose response shown in Supplementary Figure S1), with a clear differentiation between the samples and positive controls. Serum samples from three different species of monkeys (rhesus macaques, cynomolgus monkeys, and African green monkeys) showed a similar neutralizing index against 614D pseudovirus, albeit with differences between individual animals. As these results confirmed the suitability of the PBNA, we further confirmed the reproducibility of our experiments by systematically comparing the results from two independent experiments. A strong correlation (R^2^ of 0.96) suggested good reproducibility of PBNI_50_ generated in our PBNA. Such strong assay reproducibility would facilitate the further validation of this assay for evaluating the clinical samples. Our data revealed reduced neutralization of the B.1.351 SA variant with five out of six NHP challenged serum samples, despite having a higher neutralizing index for both the 614D and B.1.1.7, UK variants. Our observation of comparable neutralization capacity against 614D and B.1.1.7, UK variants, but not against the B.1.351 SA variant by the majority of (five out of six animals or 83.3% with lower neutralization against B.1.351 SA variant) serum samples from the SARS-CoV-2 WA1 challenged NHPs is not too surprising as a similar trend has been reported in the literature [29,30]. This similar profile observed between the preclinical (SARS-CoV-2-challenged NHP serum used in our experiments) and clinical samples (reported in the literature) increases the confidence in our assay system for its suitability to measure neutralizing antibodies against SARS-CoV-2. 

It is also interesting to note that five out of the six normal unexposed NHP samples tested (Koide et al, Manuscript under preparation) in our assay showed a high background neutralization index (PBNI_50_ of 100 to 250) against 614D. Only one of the NHPs showed a PBNI_50_ of < 10. As we were not sure about the reason or specificity of this background PBNI_50_ level in the unexposed NHP serum samples, we serially diluted our SARS-CoV-2-challenged NHP serum samples with high PBNI_50_ levels (> 500) to generate test samples with tiered PBNI_50_ levels (high, medium, and low) followed by evaluation in the PBNA. This experiment also enabled the measurement of linearity and a lower limit of detection of the neutralization index in the PBNA. Our experiments confirmed the linearity of detection to approximately a PBNI_50_ of 30 (the lowest PBNI_50_ tested in our assay), albeit with a high %CV at the lower end of detection. As we confirmed the sensitivity of the PBNA, high background levels (PBNI_50_ of 100 to 250) in five out of the six unexposed NHP serum samples might be due to pre-exposure to other coronaviruses in their natural environment (which might have generated neutralizing antibodies). Several previous reports regarding preexisting humoral immunity to SARS-CoV-2 in humans support this explanation [31,32]. 

Both HIV and MLV backbone-based 614D pseudoviruses behaved similarly showing similar luciferase RLU values in infected cells (Supplementary Figure S1). In addition, all three positive controls, NIBSC 20/130, Acro-SAD-S35, and Sino-40591-MM43, showed similar neutralization curves (Supplementary Figure S1B–D, respectively), further confirming the similar behavior of both the HIV and MLV backbone-based 614D pseudoviruses. HIV-based pseudotyped SARS-CoV-2 virus was used for assay characterization and testing of the NHP serum samples, whereas MLV-based virus was used for the evaluation of NIBSC 20/130 and other antibodies. 

In the literature, PBNA has been shown to correlate well with other assays measuring the neutralization antibodies, including the plaque reduction neutralization test [33], the microneutralization assay, and the ELISA assay [34]. Reports of neutralizing antibody levels in vaccinated individuals [7,35,36,37,38] and their effectiveness against various variants are emerging [39]. Recent data [35] suggests that there might be a time-dependent improvement in the neutralizing efficacy of antibodies against variants, which might be due to the expansion and evolution of antibodies in the germinal centers. However, most of the vaccines seem to elicit lower neutralizing antibody titers against the B.1.351 SA variant (beta variant). 

Our results, along with these previous reports, provide a strong justification for the implementation of PBNA as a cost-effective measure of neutralizing antibodies in the preclinical studies of vaccine candidates.

The inclusion of a universally acceptable robust positive control is essential in the evolving serological/neutralization assays for SARS-CoV-2. A recent report suggested potent neutralization of SARS-CoV-2 variants by an antibody, LY-CoV1404 [40]. However, as it is not commercially available to researchers for assay harmonization, we used NIBSC 20/130 reagent as a positive control. In our experiments, NIBSC 20/130 reagent showed impaired ability to neutralize the B.1.1.7 UK and B.1.351 SA variants. In contrast, a monoclonal antibody (Sino-40591-MM43), tested as a positive control in our experiments retained robust activity against B.1.1.7 and B.1.351 SA variants. This is one of the first reports showing the impaired ability of NIBSC 20/130 reagent to neutralize the B.1.1.7 UK and B.1.351 SA variants. Demonstration of robust activity of a commercially available monoclonal antibody (Sino-40591-MM43) against the 614D, B.1.1.7 UK, and B.1.351 SA variants is also a novel observation. It remains to be further confirmed if the neutralization activity of Sino-40591-MM43 is retained against other variants of concern, such as the B.1.617 delta and kappa variants. Nevertheless, as Sino-40591-MM43 is a commercially available monoclonal antibody, one could envision accessibility across the globe with unlimited supply (as it can be scaled up due to its the monoclonal nature). This could further enhance the global harmonization of the neutralization and serological assays against SARS-CoV-2 variants of concerns.

## 5. Conclusions

Our findings demonstrate the successful development of a robust pseudovirus-based neutralization assay against SARS-CoV-2 variants of concern, which can aid the evaluation of preclinical and clinical samples and rapidly develop effective countermeasures against SARS-CoV-2 and the variants of concern. Our results also identified a commercially available monoclonal antibody that can serve as a potential global standard in the neutralization assays against SARS-CoV-2 variants of concern.

## Figures and Tables

**Table 1:** PBNI_50_ data from independent experiments is shown in the table. A commercially available monoclonal antibody retains activity against B.1.351 SA and B.1.1.7 UK variants.

Control	614D Variant	B.1.351 SA Variant	B.1.1.7 UK Variant
**NIBSC 20/130**	4270 3541 1664	160 <800 <160	667.1 800 922
**Acro-SAD-S35**	11,511 10,502 11,164 9881	<160 <800 <160	11,192 8535 17,450
**Sino-40591-MM43**	13,973 15,706 14,182	13,828 8182 12,243	9755 16,994

## Data Availability

Data available upon request.

## Supplementary Materials

The following are available online at https://www.mdpi.com/article/10.3390/microorganisms9081744/s1, Figure S1: Similarity between SARS-CoV-2 614D pseudovirus with HIV backbone and MLV backbone.
