# Supplementary figures and images for: SARS-CoV-2 Spike Pseudoviruses: A Useful Tool to Study Virus Entry and Address Emerging Neutralization Escape Phenotypes
Source: Microorganisms. 2021 Aug 16;9(8):1744. doi: 10.3390/microorganisms9081744 (PMC8398529; doi:10.3390/microorganisms9081744)

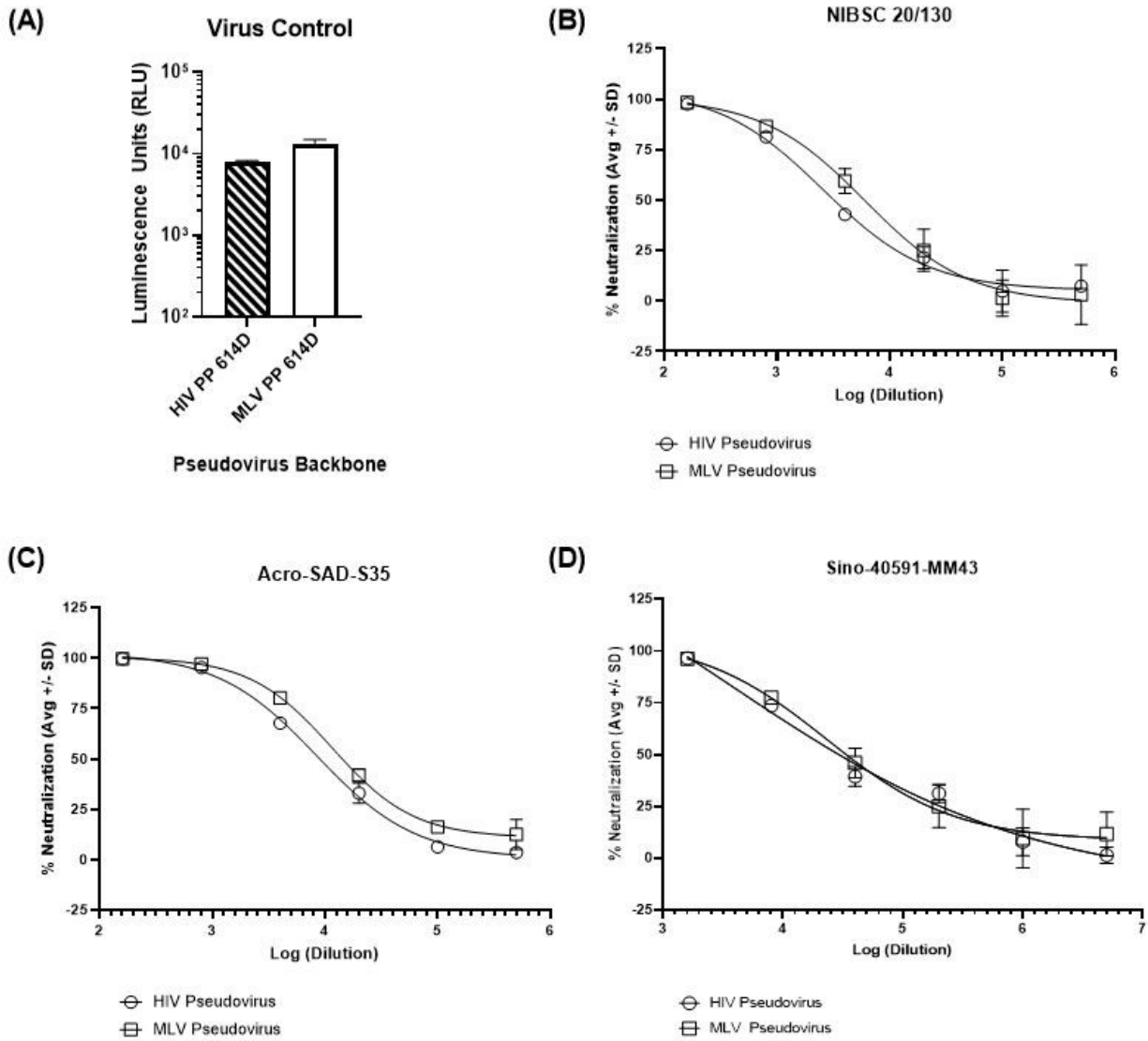

**Figure S1:** Similarity between SARS-CoV-2 614D pseudovirus with HIV backbone and MLV backbone.

Supplement: Supplementary file 1 [file microorganisms-09-01744-s001.zip › microorganisms-1321522-supplementary.pdf]
